# Long-Term Follow-Up Over 16 Years for Pituitary Hyperplasia Due to Primary Hypothyroidism With Positive Thyroid Stimulation Blocking Antibody: A Case Report

**DOI:** 10.7759/cureus.43823

**Published:** 2023-08-20

**Authors:** Muneo Kawasumi, Mitsunobu Kubota, Noriaki Matsuura, Yasuyuki Kinoshita, Atsushi Tominaga

**Affiliations:** 1 Department of Endocrinology and Diabetes, National Hospital Organization, Kure Medical Center and Chugoku Cancer Center, Kure, JPN; 2 Department of Diagnostic Radiology, National Hospital Organization, Kure Medical Center and Chugoku Cancer Center, Kure, JPN; 3 Department of Neurosurgery, Graduate School of Biomedical and Health Sciences, Hiroshima University, Hiroshima, JPN; 4 Department of Neurosurgery and Neuro-Endovascular Therapy, Hiroshima Prefectural Hospital, Hiroshima, JPN

**Keywords:** long-term follow-up, pituitary biopsy, thyroid stimulation blocking antibody, pituitary hyperplasia, primary hypothyroidism

## Abstract

Primary hypothyroidism is a known risk factor for pituitary hyperplasia, which develops symptoms due to compression of the optic chiasma and increased intracranial pressure. As pituitary hyperplasia is known to improve after levothyroxine replacement therapy, there are no reports of a long clinical course of pituitary hyperplasia due to primary hypothyroidism. We describe a case of follow-up over 16 years for pathologically diagnosed pituitary hyperplasia due to primary hypothyroidism with positive thyroid stimulation blocking antibody. Repeated enlargement and shrinkage were confirmed, but observations also suggested that the pituitary gland did not always return to normal size.

## Introduction

Primary hypothyroidism leads to pituitary hyperplasia via loss of negative feedback. Low thyroid hormone levels cause excess production of thyroid stimulating hormone (TSH)-releasing hormone (TRH) from the hypothalamus. Excess TRH is thought to stimulate thyrotrophs and promote their proliferation, leading to pituitary hyperplasia [1]. Pituitary hyperplasia occurs under various physiological and pathological conditions and is common in clinical practice [2-5]. The incidence of pituitary hyperplasia due to hypothyroidism has been reported to range from 25% to 81% [6]. It is typically diagnosed through imaging, and can occasionally be difficult to differentiate from TSH-producing adenomas on CT or MRI. In addition, pituitary hyperplasia is often diagnosed based on thyroid function and clinical course after thyroid hormone replacement therapy [7].

We herein report a case of pathologically diagnosed pituitary hyperplasia due to primary hypothyroidism with positive thyroid stimulation blocking antibody (TSBAb). In daily practice, we encountered an 18-year-old woman with suspected pituitary apoplexy. A follow-up MRI showed dramatic shrinkage of the pituitary gland with levothyroxine replacement therapy. Thyroid function and pituitary gland size were monitored for approximately 16 years.

This article was previously posted to the preprint server Research Square on April 6, 2023.

## Case presentation

**Figure 1:**
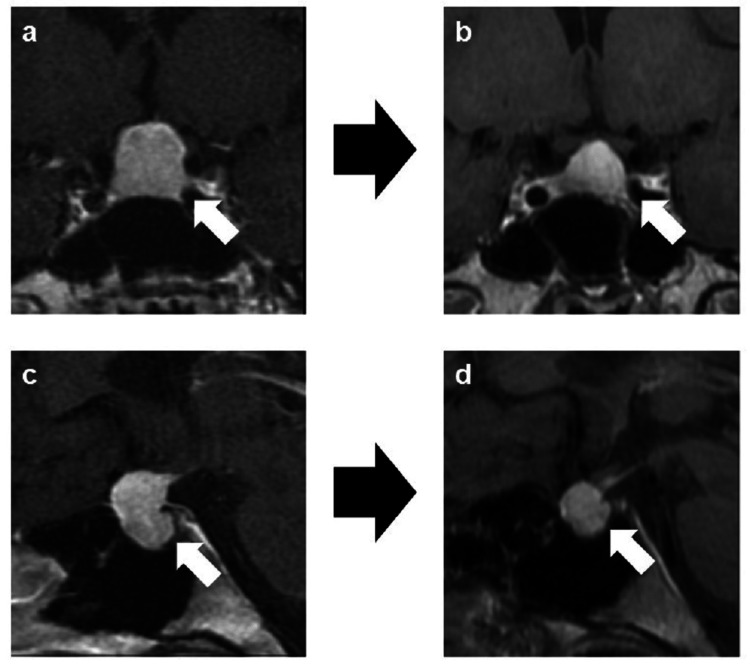
MRI scan imaging of the pituitary gland. Coronal contrast-enhanced T1-weighted (a, b) and sagittal contrast-enhanced T1-weighted (c, d) MRI scans at the first visit to our hospital (a, c) and 10 months after administration of levothyroxine (b, d). After 10 months of levothyroxine replacement therapy, a follow-up MRI showed dramatic pituitary shrinkage of approximately 50% to a tumor size of 16.79×11.28×12.59 mm (white arrows). MRI: magnetic resonance imaging

An 18-year-old female experienced headache, double vision, and bitemporal hemianopia and was transported to our hospital in an ambulance. She had no past medical history. A large suprasellar tumor measuring 19.2×13.67×18.96 mm and compressing the optic chiasma was detected on MRI, and pituitary apoplexy was suspected. Hemorrhage in the tumor was not confirmed (Figure 1).

She was transferred to Hiroshima University Hospital for the purpose of neurosurgical scrutiny. On questioning, she gave a history of weight gain and constipation. She had delayed deep tendon reflexes, dry skin, and lower leg edema. Her serum levels of TSH and prolactin (PRL) were elevated, whereas free triiodothyronine (FT3) and free thyroxine (FT4) levels were reduced (Table 1). Serum levels of cortisol, adrenocorticotropic hormone (ACTH), and growth hormone (GH) were normal and thyroglobulin, thyrotropin receptor antibody (TRAb), and TSBAb were elevated, respectively. Later examination revealed anti-thyroid peroxidase antibody (TPOAb) and anti-thyroglobulin antibody (TgAb) were also elevated.

**Table 1:** Significant laboratory results As the phase of the sex cycle at the time of blood collection was unknown, it is difficult to provide a reference range for sex hormones. TPOAb and TgAb were measured in X+9. Radioimmunoassay was used to detect and quantify the thyroid stimulation-blocking antibody. FT3: free triiodothyronine; FT4: free thyroxine; TSH: thyroid stimulating hormone; PRL: prolactin; ACTH: adrenocorticotropic hormone; LH: luteinizing hormone; FSH: follicle stimulating hormone; GH: growth hormone; TRAb: thyrotropin receptor antibody; TSBAb: thyroid stimulation blocking antibody; TPOAb: anti-thyroid peroxidase antibody; TgAb: anti-thyroglobulin antibody

	Patient's blood level	Reference range
FT3 (pg/mL)	<0.7	2.3 - 4.7
FT4 (ng/dL)	0.1	1.1 - 1.9
TSH (μIU/mL)	844.5	0.31 - 3.15
PRL (ng/mL)	80.8	0.0 - 26.3
Cortisol (μg/dL)	9.3	4.5 - 18.0
ACTH (pg/mL)	13.4	7.4 - 55.7
LH (mIU/mL)	0.3	
FSH (mIU/mL)	4.5	
Estradiol (pg/mL)	304	
GH (ng/mL)	0.55	0.0 - 3.10
Thyroglobulin (ng/mL)	39.3	0.0 -32.7
TRAb (IU/L)	1.5	0.0 - 0.9
TSBAb (%)	83.7	0.0 - 45.6
TPOAb (IU/mL)	93.1	16>
TgAb (IU/mL)	50.8	28>

Following her imaging and endocrine data, pituitary hyperplasia due to primary hypothyroidism was diagnosed clinically and levothyroxine replacement therapy was started (50 µg/day). The loading dose was increased to 200 µg/day. Twelve days later, a repeat MRI showed shrinkage of the pituitary gland to 19.08×12.20×16.71 mm, which decreased the compression of the optic chiasma; thyroid function laboratory data was also improved with a TSH of 341.3 µIU/mL, FT3 of 1.9 pg/mL, and FT4 of 0.9 ng/dL. Three months after visiting our hospital, subjective symptoms such as headache, double vision, and bitemporal hemianopia disappeared. Follow-up MRI revealed little change in the tumor size, at 19.63×12.43×15.71 mm.

**Figure 2:**
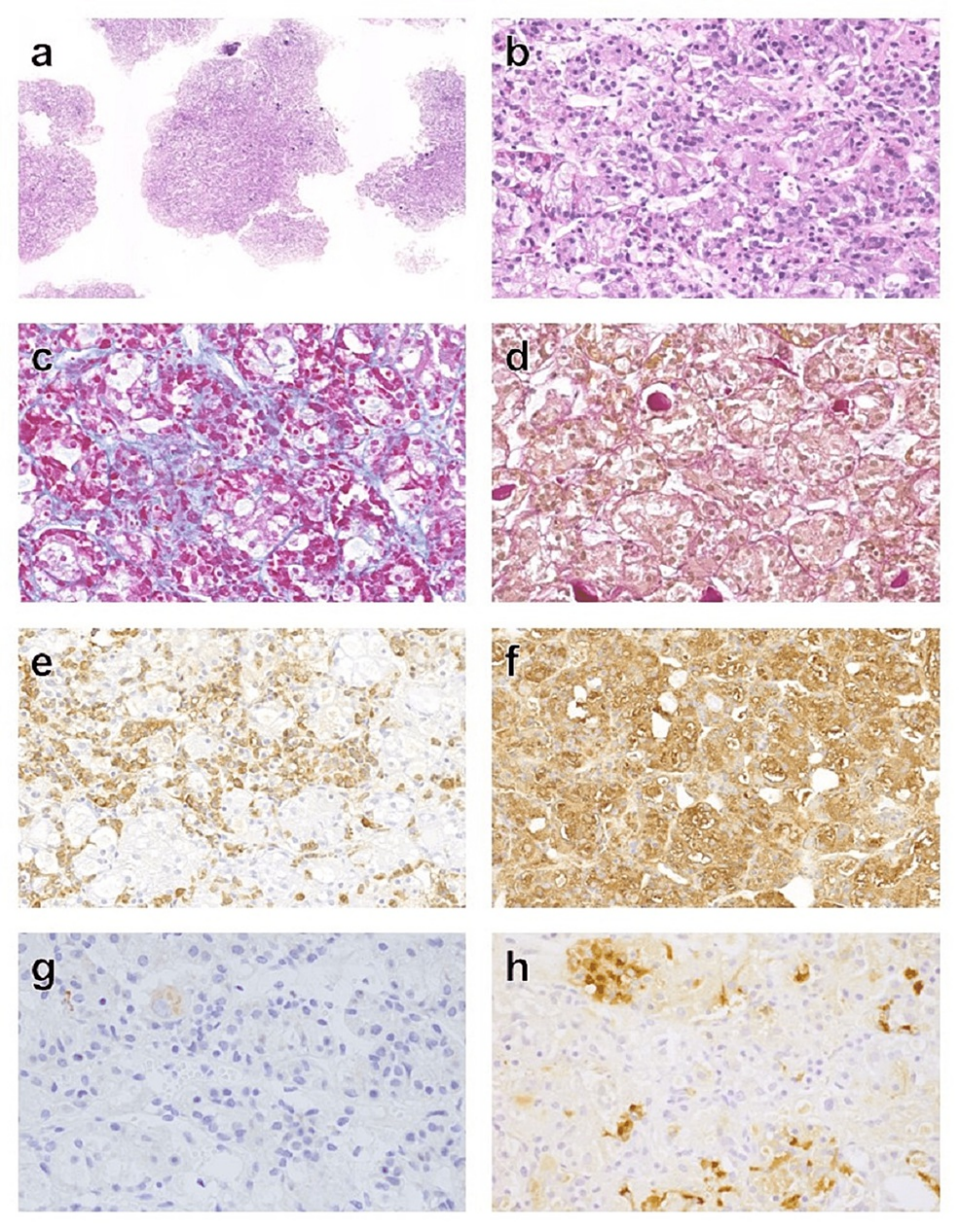
Histopathological and immunohistochemical staining of the pituitary gland. HE (a), HE (b), Azan (c), PAS-OG (d), GH (e), TSH (f), Ki-67 (g), and PRL (h). Original magnifications, ×5 (a) and ×40 (b-h). The following primary antibodies were used: GH (Ventana 760-2804), TSH (DAKO M3503), Ki-67 (Ventana 790-4286), and PRL (DAKO A0569). HE: hematoxylin-eosin; PAS-OG: periodic acid-Schiff-orange G; GH: growth hormone; TSH: thyroid stimulating hormone; PRL: prolactin

For the purpose of ruling out invasive pituitary tumors, the lower one-fourth of the pituitary gland was biopsied by a transsphenoidal endoscopic approach. Histopathological examination showed pituitary hyperplasia. The small acinar proliferation of anterior pituitary cells was evident, dominated by chromophobe epithelioid cells. The specimens showed an absence of nuclear mitoses and necrosis, and the reticulin acinar pattern was preserved. Immunohistochemically, most cells were positive for TSH and negative for Ki-67 (Figure 2).

The results of the insulin, TRH, and LH-releasing hormone (LHRH) loading tests performed after endoscopic biopsy are shown in Table 2. Pituitary dysfunction was not observed. The levothyroxine loading dose was gradually increased to 150 µg/day. After 10 months of levothyroxine replacement therapy, a follow-up MRI showed dramatic pituitary shrinkage of approximately 50% to a tumor size of 16.79×11.28×12.59 mm, although this size was influenced by biopsy (Figure 1). Laboratory evaluation also showed further improvement in thyroid function with a TSH of 34.68 µIU/mL, FT3 of 2.1 pg/mL, and FT4 of 0.9 ng/dL.

**Table 2:** Combined pituitary stimulation test (insulin, TRH, LHRH). LH: luteinizing hormone; FSH: follicle stimulating hormone; GH: growth hormone; TSH: thyroid stimulating hormone; PRL: prolactin; TRH: TSH-releasing hormone; LHRH: LH releasing hormone

	Basal	Peak	(min)
LH (mIU/mL)	<0.1	17.7	(30)
FSH (mIU/mL)	2.3	4.3	(60)
GH (ng/mL)	0.15	21.03	(60)
TSH (μU/mL)	322.5	1577	(60)
PRL (ng/mL)	28.2	227.6	(30)
Cortisol (μg/dL)	7.7	21.5	(60)

**Figure 3:**
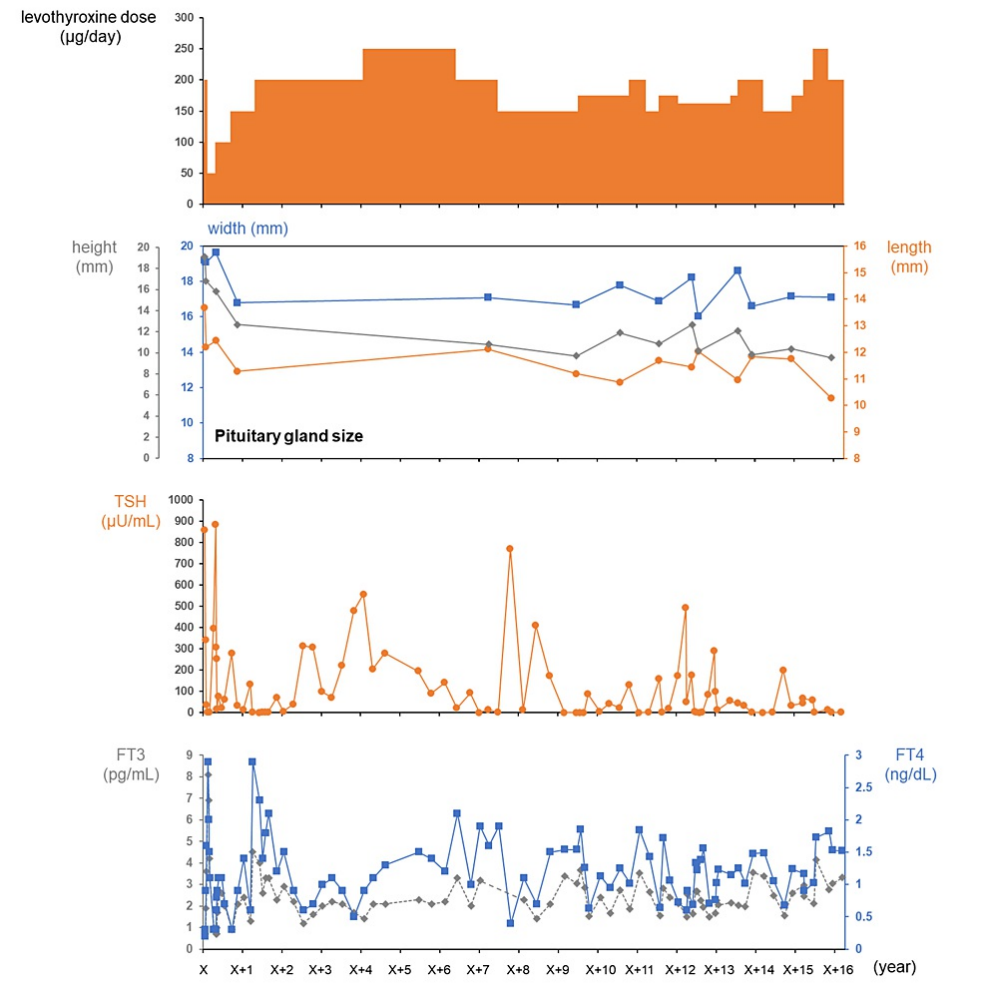
Clinical course. Serum FT3, FT4, and TSH were initially measured by electro-chemiluminescence immunoassay (Roche Diagnostics Inc., Tokyo, Japan). The measurement method was changed to chemiluminescent immunoassay (Abbott Japan LLC., Chiba, Japan) as of May 29, X+9. FT3: free triiodothyronine; FT4: free thyroxine; TSH: thyroid stimulating hormone

Afterward, oral levothyroxine was continued. The prescribed dose of levothyroxine during a clinical course of approximately 16 years is shown in Figure 3. However, the patient often forgot to take her medication; as such, the actual oral dose is uncertain. Thyroid function deteriorated many times over the clinical course. At each time point, the patient was found in the medical interview to not be taking her levothyroxine tablets and was urged to improve her medication adherence.

**Figure 4:**
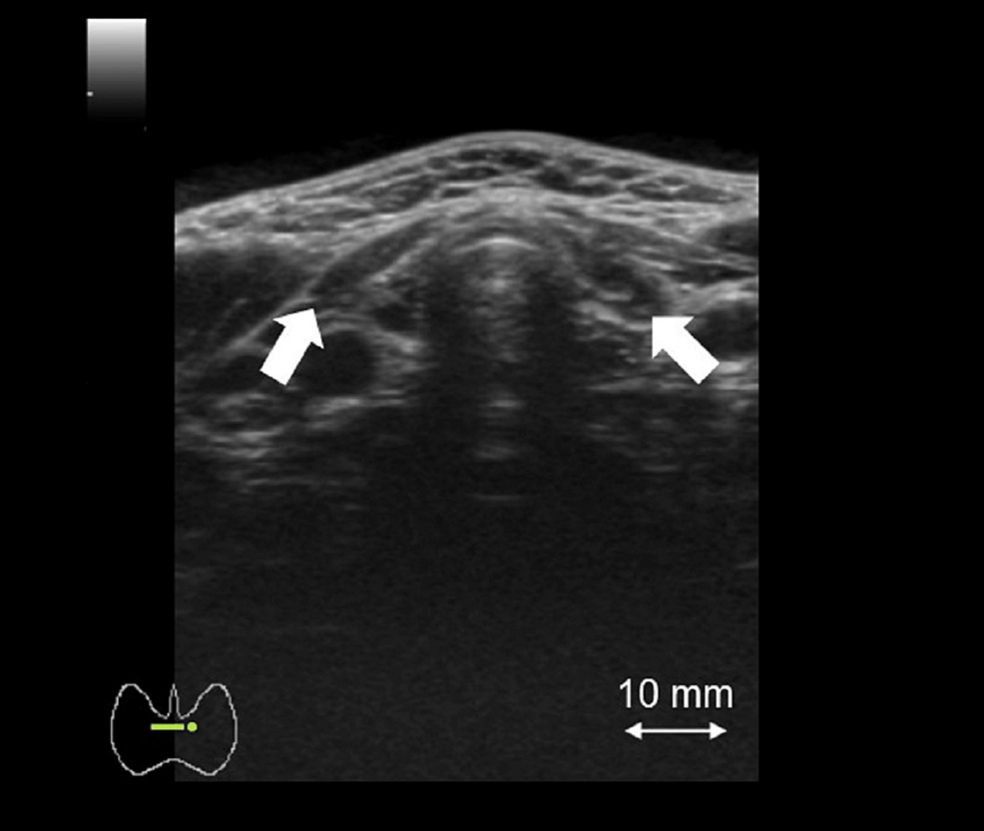
Thyroid ultrasonography. Ultrasonographic findings of the thyroid gland demonstrating the remarkable shrinkage and irregular surface and heterogeneous low-level internal echoes without high Doppler blood flow at nine years after initial presentation (white arrows, right lobe 44×7.4×7.1 mm, left lobe 46×5.6×6.6 mm, isthmus thickness 1.4 mm).

**Figure 5:**
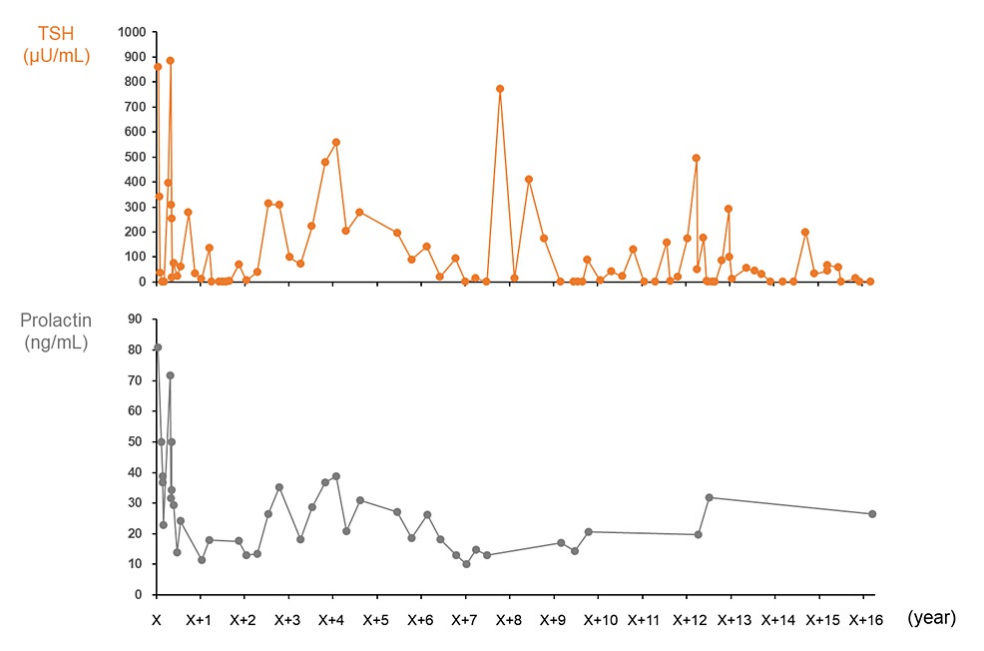
The changes in TSH and PRL levels over time. TSH: thyroid stimulating hormone; PRL: prolactin

Nine years after her initial presentation, the patient came to visit our hospital of her own accord. At the time of our consultation, her thyroid function was well controlled with a TSH of 0.25 µIU/mL, FT3 of 3.39 pg/mL, and FT4 of 1.54 ng/dL under a loading dose of 150 µg/day. Follow-up MRI of the pituitary also showed a decrease in height with no change in the width and length, with dimensions 16.67×11.19×9.64 mm (Figure 3). Ultrasonographic findings of the thyroid gland revealed a remarkable shrinkage and irregular surface and heterogeneous low-level internal echoes without high Doppler blood flow, compatible with autoimmune atrophic thyroiditis due to TSBAb (Figure 4). Subsequently, the size of the pituitary was evaluated by MRI once a year. Reversible pituitary enlargement was shown, although transient changes in thyroid function had no correlation with pituitary gland size (Figure 3). In the clinical course, there was a correlation between TSH and PRL levels (Figure 5). At approximately 16 years after the initiation of levothyroxine replacement therapy, the patient had no symptoms associated with pituitary hyperplasia, including headache and visual field disorder.

## Discussion

Over approximately 16 years, we followed thyroid-related hormones and pituitary size in a patient with a definitive diagnosis of pituitary hyperplasia due to primary hypothyroidism with positive TSBAb. To the best of our knowledge, there has not been any report describing a long clinical course of pituitary hyperplasia due to primary hypothyroidism diagnosed by pituitary biopsy findings. This case shows that although pituitary gland volume is known to decrease after levothyroxine replacement therapy, the pituitary gland may not return to normal size after the appearance of profound pituitary hyperplasia.

A previous observational study reported a correlation between serum TSH levels and the frequency of pituitary enlargement. Specifically, pituitary enlargement was observed in 70% of patients with TSH levels greater than 50 μIU/mL and in 84% with TSH levels greater than 100 μIU/mL [6]. In addition, pituitary volume in patients with PRL-producing pituitary adenomas is generally known to correlate with serum PRL level [8]. To the best of our knowledge, there is no known correlation between pituitary volume and serum TSH level in patients with pituitary hyperplasia due to hypothyroidism. In our case, such a correlation was also not observed (Figure 3). TSH is regulated by TRH stimulation in the hypothalamus-pituitary-thyroid axis; accordingly, serum TSH levels are thought to be associated not only with pituitary volume but also with factors including TRH level in the pituitary portal vein, oral dose of levothyroxine, and the percentage of thyrotrophs with high TSH secretion ability among all adenohypophysis cells.

In our case, no symptoms associated with pituitary enlargement were confirmed during the clinical course after levothyroxine replacement therapy. However, the repeated enlargement and shrinkage of the pituitary gland means that the occasional lack of levothyroxine intake exacerbated pituitary enlargement. The patient’s medication non-adherence makes it difficult to accurately assess the actual dose by medical interview alone. It is important to regularly evaluate thyroid function in addition to performing imaging tests in the clinical course of pituitary hyperplasia due to primary hypothyroidism. Given that longstanding TRH stimulation causes pituitary hyperplasia, TRH measurement is important for controlling pituitary volume; however, it is difficult to acquire. TSH and PRL levels can be latent indicators for controlling pituitary volume because they are downstream of TRH. Therefore, serum TSH and PRL levels are thought to help in determining an appropriate daily oral dose of thyroid hormone.

There are two types of autoimmune thyroiditis, autoimmune goitrous Hashimoto’s thyroiditis and autoimmune atrophic thyroiditis [9]. Some patients with atrophic thyroiditis are confirmed to produce TSBAb, which causes hypothyroidism due to TSH resistance via inhibition of the TSH-stimulated cyclic adenosine monophosphate (cAMP) response [10]. Low uptake of 123I has been reported in patients with primary hypothyroidism accompanied by TSBAb and Graves’ disease for whom the hypothyroidism developed after the appearance of TSBAb [11, 12]. In our case, TSBAb activity was high in addition to primary hypothyroidism. Low levels of T3 and T4 increase TRH secretion from the hypothalamus via a loss of negative feedback. Excess TRH is thought to stimulate thyrotrophs and promote their proliferation, which leads to pituitary hyperplasia. Therefore, TSBAb is assumed to factor into the failure to suppress TRH secretion and normalize pituitary size.

Pituitary hyperplasia due to primary hypothyroidism is known to be responsive to thyroid hormone replacement therapy [13]. In our case, the pituitary gland volume was reduced by approximately 50% after 10 months of levothyroxine replacement therapy. However, the molecular mechanism by which pituitary gland volume shrinks with thyroid hormone replacement therapy is unknown. The pituitary gland volume in prolactin-producing adenomas is reportedly reduced by the administration of bromocriptine, a dopamine agonist [14]. Although past reports suggested the tumor-size-reducing effect of bromocriptine occurs through cellular necrosis with infarction [15-17], transmission electron microscopy revealed the molecular mechanism to be based on shrinkage of cell volume rather than a decrease in cell number due to cellular necrosis [18, 19]. Here, thyroid hormone replacement therapy may have caused the pituitary gland volume to regress as a result of volume shrinkage of thyrotrophs due to low stimulation of TRH followed by a decrease in the ability to produce TSH.

The pituitary volume did not converge to normal size during the clinical course of about 16 years. This is thought to be mainly due to the patient’s non-adherence concerning levothyroxine intake, where frequent non-adherence provoked excessive TRH production through the loss of negative feedback in the hypothalamus-pituitary-thyroid axis, which led to pituitary hyperplasia. That is why the pituitary gland enlarged and contracted repeatedly. However, based on the fact that shrinkage of cell volume has been reported as the molecular mechanism of pituitary volume reduction [18, 19], the pituitary gland may not return to normal size after the appearance of profound pituitary hyperplasia.

## Conclusions

We reported a case of long-term follow-up over 16 years for pathologically diagnosed pituitary hyperplasia due to primary hypothyroidism with positive thyroid stimulation blocking antibody. This report highlights the fact that the pituitary volume may not converge to a normal size after the appearance of profound pituitary hyperplasia. The patient’s non-adherence with levothyroxine intake caused a reversible pituitary enlargement, leading to symptoms due to compression of the optic chiasma and increased intracranial pressure. In the case of poor medication adherence, medication guidance is important to be provided assiduously for the prevention of profound pituitary hyperplasia, while using TSH and PRL levels as indicators.
